# Which One Is More Important, Obesity or Cardio Metabolic Risk Factors?

**DOI:** 10.5812/ijem.7963

**Published:** 2012-12-21

**Authors:** Farhad Hosseinpanah

**Affiliations:** 1 Obesity Research Center, Research Institute for Endocrine Sciences, Shahid Beheshti University of Medical Sciences, Tehran, IR Iran

**Keywords:** Obesity, Cardio Metabolic

Editorial

Obesity defined as a BMI of 30 or above is a major health hazard, which is obviously linked to dyslipidemia, hypertension and type2 diabetes. However, clustering of CVD risk factors is different across the continuum of body mass index (BMI) leading to recognition of phenotype subgroups of obesity. Accordingly since the 1980s two well-known phenotypes of obesity have been described in medical literature i.e. “Metabolically obese but normal weight” (MONW) and “metabolically healthy but obese” (MHO) (1). Among American adults in the National Health and Nutrition Examination Survey (NHANES; 1999 to 2004), 23.5% of normal-weight adults were metabolically abnormal, whereas 51.3% of overweight adults and 31.7% of obese adults were metabolically healthy (2). Moreover few studies reported an increasing trend of MNOW. For instance a dramatic 4-fold increase in prevalence of metabolic syndrome has been reported among normal weight Tehranian adults during 6.6 years of follow up (3). There are controversial findings regarding the independent role of obesity in the development of CVD and all-cause mortality. Some studies have shown that obesity, regardless of the presence or absence of Metabolic syndrome and IR, was associated with a higher risk for Cardio metabolic comorbidities (4). While, others have demonstrated that BMI, and other anthropometric indices, whether assessed singly or in combination, do not importantly improve cardiovascular disease risk prediction when additional information is available for systolic blood pressure, history of diabetes, and lipids (5). Accordingly, considering the different outcomes for the phenotypes, importance of metabolic abnormalities for predicting CVD events in comparison with weight status per se emerges as an important question. In fact it seems that some kinds of interaction exist between obesity and metabolic risk factors in the development of CVD outcomes. Obesity-related phenotypic characteristics and CVD outcomes have been investigated in several epidemiological studies yielding some conflicting results (6-12). The majority of studies have reported an increased risk of CVD in MNOW compared to MHO. Within the framework of Tehran Glucose and Lipid Study (TLGS), during a mean of 8.1 years of follow up, among 6,215 subjects aged >30 years free of CVD at baseline, we showed that, compared to normal weight subjects without dysmetabolic aspects, multivariate-adjusted hazard ratios for CVD events were 2.10 (95% CI 1.36 to 3.26) and 1.07 (95% CI 0.59 to 1.96) in normal weight subjects with dysmetabolic aspects and obese subjects without dysmetabolic aspects respectively (9). A recent large and well conducted study by Hamer and Stamatakis also reported that metabolically healthy obese participants were not at increased risk of CVD and all-cause mortality following an average 7 years of follow up. Moreover metabolically unhealthy obese participants were at elevated risk of all-cause mortality compared with their metabolically healthy obese counterparts (HR 1.72,95% CI 1.23–2.41) (10). However, other reports provided contradictory results regarding the benign nature of MHO (11, 12). Johan Arnlov et al. investigated associations between cardiovascular disease and death in middle-aged men during more than 30 years of follow-up and found that men with metabolic syndrome had an increased risk of cardiovascular events, regardless of BMI status. However overweight and obese participants without metabolic syndrome or without IR also had an increased risk of cardiovascular events and total death compared with normal-weight men without metabolic syndrome or insulin resistance (12). These studies differed from each other in terms of varying definitions for the phenotypes, the types of outcomes and ascertainment methods used and length of follow up. The positive association between MHO and CVD outcomes observed with long term follows up, can be explained by possible metabolic deterioration of obese subjects who are free of metabolic abnormalities at baseline. However the natural course of metabolic syndrome incidence in healthy obese phenotype has not been investigated in long term prospective studies (13, 14). Taken together, there is no doubt regarding the increased risk of CVD in MNOW but further studies are needed to answer the question whether MHO is associated with CVD or not. This unanswered question has led to different recommendations by national and international guidelines in terms of the value of anthropometric indices for prediction of CVD outcomes (15-18). Moreover, a better understanding of MHO prognosis can help the physicians to identify those overweight or obese subjects who should be candidates for early intervention. Until we have definitive answers, it seems reasonable to put more weight on metabolic risk factors as compared to anthropometric measures especially BMI.
